# Predicting the dynamic distribution of *Sphagnum* bogs in China under climate change since the last interglacial period

**DOI:** 10.1371/journal.pone.0230969

**Published:** 2020-04-06

**Authors:** Mingyang Cong, Yueyue Xu, Luyan Tang, Wenjing Yang, Minfei Jian

**Affiliations:** 1 Analytical & Testing Center, Jiangxi Normal University, Nanchang, Jiangxi Province, China; 2 Jiangxi Provincial Key Lab of Protection and Utilization of Subtropical Plant Resources, Jiangxi Normal University, Nanchang, Jiangxi Province, China; 3 School of Economics & Management, Nanchang University, Nanchang, Jiangxi Province, China; 4 College of Life Science, Guizhou Normal University, Guiyang, Guizhou Province, China; 5 Key Laboratory of Poyang Lake Wetland and Watershed Research, Ministry of Education, Jiangxi Normal University, Nanchang, Jiangxi Province, China; Hellenic Agricultural Organization - Demeter, GREECE

## Abstract

*Sphagnum* bogs possess irreplaceable ecological and economic value, and they are scarce in China, with a fragmented distribution. Based on 19 high-resolution bioclimatic environmental datasets and 71 bog center point locations, we employed a maximum entropy model (MaxEnt) to reconstruct and predict the spatial-temporal geographical distribution patterns of *Sphagnum* bogs from the last interglacial (LIG) period to two typical CO_2_ representative concentration pathway scenarios (RCP2.6, RCP8.5) in the future. We further computed the migratory paths of the distribution center points. Finally, a jackknife test was used to uncover the crucial environmental factors restricting the geographical distribution of the bogs. Our data indicated that the MaxEnt niche model had a high simulation precision with an area under the ROC curve value of 0.957. Spatially, the suitable bog habitats are currently centralized in northeastern China, including the Greater Khingan Mountains, the Lesser Khingan Mountains, and the Changbai Mountains, as well as peripheral areas of the Sichuan Basin. Temporally, the contours of *Sphagnum* bogs were similar to the present and rendered from the last glacial maximum (LMG) period, and had much more total area than the current. The total area in LIG was nearly the same as the current because of the similar climate. It was worth noting that there would be a reduction of the total area in the future. Loss of area occurred at the edges of bogs, especially under RCP8.5. The distribution center of bogs will shift to the northwest in the immediate future. The precipitation of driest month, the mean temperature of warmest quarter and the precipitation of warmest quarter were identified as crucial climatic factors affecting the distribution of *Sphagnum* bogs. Overall, our research provides scientific evidence for the long-term protection and effective management of these rare, precious natural resources and suggestions for in situ conservation.

## Introduction

Climate change is an indisputable fact. Repeated alternations between ice ages and interglacial periods in the Late Quaternary, including the last interglacial period (LIG, ~120,000–140,000 years) and the last glacial maximum (LGM, ~21,000 years), caused the formation of the modern global distribution patterns of vegetation [1]. Extensive studies have proven that suitable areas for vegetation shift and migrate along with global climate changes [2,3]. Unfortunately, similar studies have been performed for forest [4] and grassland [5] ecosystems on a large scale, but little attention has been paid to peatland ecosystems.

Peatlands are vital parts of natural wetland ecosystems that are special and irreplaceable; peatlands also possess substantial economic value because they are reserves for precious mineral coal resources. As reported earlier, despite accounting for 3% of the total land area, northern peatlands sequester as much as approximately one-third of the world’s carbon supply and play a considerable role in the global carbon cycle [6–8]. Peatlands are fragile ecosystems, and the negative effects of their degeneration or damage are cause for concern. Moreover, damage to peatlands may intensify greenhouse effects by causing a sharp rise in carbon emissions, leading to irreversible water loss and soil erosion and making coal resources unsustainable.

Peatlands are categorized as bogs and fens, with the dominant species being mosses and herbaceous plants, respectively. Among them, *Sphagnum* plants evolved more than 200 million years ago, and cold and damp climates gave rise to *Sphagnum* bogs, a type of peatland. As dominant species, *Sphagnum* plants are sensitive to climate change due to their oversimplified gametophytes [9,10]. China is located in the East Asian monsoon zone, where the earth’s climatic environment is predicted to change the most. A previous study shows that the distributional area of wetlands reduces more quickly than that of other ecosystems [11]. Hence, it is inferred that climate change is likely to affect the distribution of *Sphagnum* bogs in China. Most studies on the response of bogs to global change have concentrated on microdomains, such as the carbon cycle [12]. However, little is known about the spatio-temporal distribution patterns and suitable areas for *Sphagnum* bogs in China with ongoing climate change. As stated above, this lack of knowledge is detrimental to the value assessment and resource management of *Sphagnum* bogs. Therefore, it is urgent to preserve *Sphagnum* bog habitats in the face of climate change.

Recently, the prediction of potential distribution areas under climate change using species distribution models (SDMs) has become a research hot spot. The method is to combine SDMs and ArcGIS to identify areas with high ecological stability in the process of climate change [13]. SDMs identify relationships between the known occurrence of a species (presence or presence/absence data) and environmental data, and use these relationships to make predictions for all unsampled areas in the study region [14,15]. Most studies have been directed at invasive [16], endangered [17], medicinal [18,19], bioenergy [20] and ornamental plants [21]. Nevertheless, studies on suitable areas for *Sphagnum* bogs and their relationships with climate change in China are weak.

Many studies have proven that the maximum entropy model (MaxEnt) predicts potential distribution areas well and is broadly applied in conservation, biology, ecology and other fields [22–24]. Earlier similar studies predicting the distribution of *Grimmia* Hedw. in Mexico and *Hypopterygium tamarisci* (Sw.) Brid. ex Müll. Hal. in Central and South America indicate that MaxEnt has a good correspondence with collected site data [25,26]. Furthermore, MaxEnt can also be projected into the Late Quaternary (containing LIG and LGM) by integrating climatic data. Meanwhile, applying MaxEnt to glacial and interglacial periods and converting SDM into a habitat resistance model allows the use of a minimal cost path to identify species migration routes [13, 27].

For these reasons, in this study, we address the following scientific issues. (1) What has the impact of climate change been on the distribution of *Sphagnum* bogs over time? (2) What are the migration routes of the central distribution points? (3) What are the leading climatic factors limiting the geographical distributions of *Sphagnum* bogs? This article revealed the spatio-temporal distribution patterns and dynamic changes of *Sphagnum* bogs in China. The results not only provide a scientific basis for the sustainable development of natural resources but also lay a foundation for strengthening the value assessment and scientific protection of peatlands in China.

## Materials and methods

### Center points of *Sphagnum* bogs

To collect the longitude and latitude for *Sphagnum* bogs’ central points across China, we principally consulted *Swamps in China* [28] and *Marshes in China* [29] and then extracted occurrence data from the literature. For the records lacking specific geographic coordinates, we used Google Earth (https://earth.google.com/web/) to conduct toponymal geocoding. Altogether, 71 unrepeated presence records were collected (46 literature records and 25 vectorization records) and are shown in the Supporting Information S1 Dataset.

### Environmental parameters

Generally, nineteen bioclimatic variables related to precipitation and temperature are used to model and predict species distribution. The collinearity between variables in the SDMs may lead to over-fitting phenomenon. However, Xiao Feng et al. reports that the strategy of excluding highly correlated variables has little impact because Maxent accounts for redundant variables [30]. Besides, the nineteen environmental variables are regular bioclimatic variables, and the growth and distribution of *Sphagnum* plants are susceptible to precipitation and temperature. Therefore, all the nineteen bioclimatic environmental variables r participated in predicting the pattern of *Sphagnum* bogs. The codes used above for variables are listed in Table 1.

**Table 1:** Environment variables used for predicting the geographical distribution of *Sphagnum* bogs.

Codes	Environment variables
**Bio1**	Annual mean air temperature
**Bio2**	Mean diurnal air temperature range
**Bio3**	Isothermality (Bio2/Bio7)(*100)
**Bio4**	Temperature seasonality (standard deviation *100)
**Bio5**	Max temperature of warmest month
**Bio6**	Min temperature of coldest month
**Bio7**	Temperature annual range (Bio5-Bio6)
**Bio8**	Mean temperature of wettest quarter
**Bio9**	Mean temperature of driest quarter
**Bio10**	Mean temperature of warmest quarter
**Bio11**	Mean temperature of coldest quarter
**Bio12**	Annual precipitation
**Bio13**	Precipitation of wettest month
**Bio14**	Precipitation of driest month
**Bio15**	Precipitation seasonality (coefficient of variation)
**Bio16**	Precipitation of wettest quarter
**Bio17**	Precipitation of driest quarter
**Bio18**	Precipitation of warmest quarter
**Bio19**	Precipitation of coldest quarter

### Historical and future climate scenarios

In addition to current data, historical (LIG, LGM) and future (2050: 2041–2060, 2070: 2061–2080) climate scenarios were also needed. All climate scenario data were calculated by the CCSM model (BCC-CSM1-1) [31], which was developed by the National Center for Atmospheric Research from the WorldClim database (https://www.worldclim.org/). The future climate scenarios included two typical CO_2_ representative concentration pathways (RCPs), namely, RCP2.6 and RCP8.5, which represent two scenarios of the future global average temperature increase over the current average, with a minimum increase of 1.0 °C and a maximum increase of 2.0 °C, respectively. Next, the future climate datasets were applied to the four combined scenarios: RCP2.6–2050, RCP2.6–2070, RCP8.5–2050, and RCP8.5–2070.

### Map vector data and software

A 1:1000000 administrative regionalization map of China was downloaded from the National Catalogue Service for Geographic Information (http://www.webmap.cn/main.do?method=index). ArcGIS10.3 was obtained from the Geographic Information System platform developed by the Environmental Systems Research Institute company in the United States. MaxEnt 3.3.3 k software was obtained from the Princeton University website (http://www.cs.princeton.edu/~schapire/maxent/) [22].

### Predicting the dynamic distribution pattern of *Sphagnum* bogs

The receiver operating characteristic (ROC) curve was employed to test prediction accuracy by judging the AUC (area under the ROC curve) value (0~1) [24]; the prediction was judged to be perfect if the AUC value was 1 [32]. After importing the distribution and environmental datasets to MaxEnt 3.3.3 k, we calculated the contribution rate of each environmental variable with a jackknife test [33] and set the other parameters to default values. Simultaneously, we repeated operation ten times by cross-validation and then output the ASCII grid layer with the largest AUC value. Because it is not affected by thresholds that are insensitive to the incidence of species, the AUC value is acknowledged as the best evaluation indicator [34].

Subsequently, the arithmetic results from MaxEnt were loaded into ArcGIS10.3 to carry out suitability classification and visualization and thereby generate the potential distribution of *Sphagnum* bogs. It was critical to choose an appropriate threshold when converting the continuous species suitability prediction results into a Boolean classification of suitable and unsuitable habitats. The sensitivity-specificity sum maximization approach was verified to be superior to other threshold division methods.

### Calculating the shifts in the distribution area

After modeling the current suitable habitat area for *Sphagnum* bogs, changes in the potential distribution areas were calculated. Future climate datasets were used to carry out the modeling and forecasting for calculating future suitable habitat areas. We cross-checked future suitable habitat areas against the current distribution to identify regions that were (i) gains, (ii) unchanged and (iii) losses. We then calculated the area of the regions identified in (i)-(iii).

### The core distributional shifts in *Sphagnum* bogs

For the sake of further exploring the dynamic migration paths of *Sphagnum* bogs, we calculated the centroids of *Sphagnum* bogs from their historical distribution to their future distribution by using a Python-based SDM toolbox [35]. The analysis concentrated the species distribution into an independent central point and created a vector file depicting the magnitude and direction of changes over time [36]. We observed the distributional shifts by tracking the centroid changes among different SDMs.

### Assessment of key environmental factors

The operation principle of the jackknife method is to create a series of new models by using a variable or excluding a variable in turn. Then, we compared the model results to regularized training data, testing gains and differences in AUC values among the models to assess the importance of the environment variables.

The step-by-step laboratory protocols were deposit in protocols.io (https://www.protocols.io/view/protocols-for-predicting-sphagnum-bogs-distributio-bbtxinpn).

## Results

### Accuracy evaluation of the MaxEnt niche model

**Fig 1:**
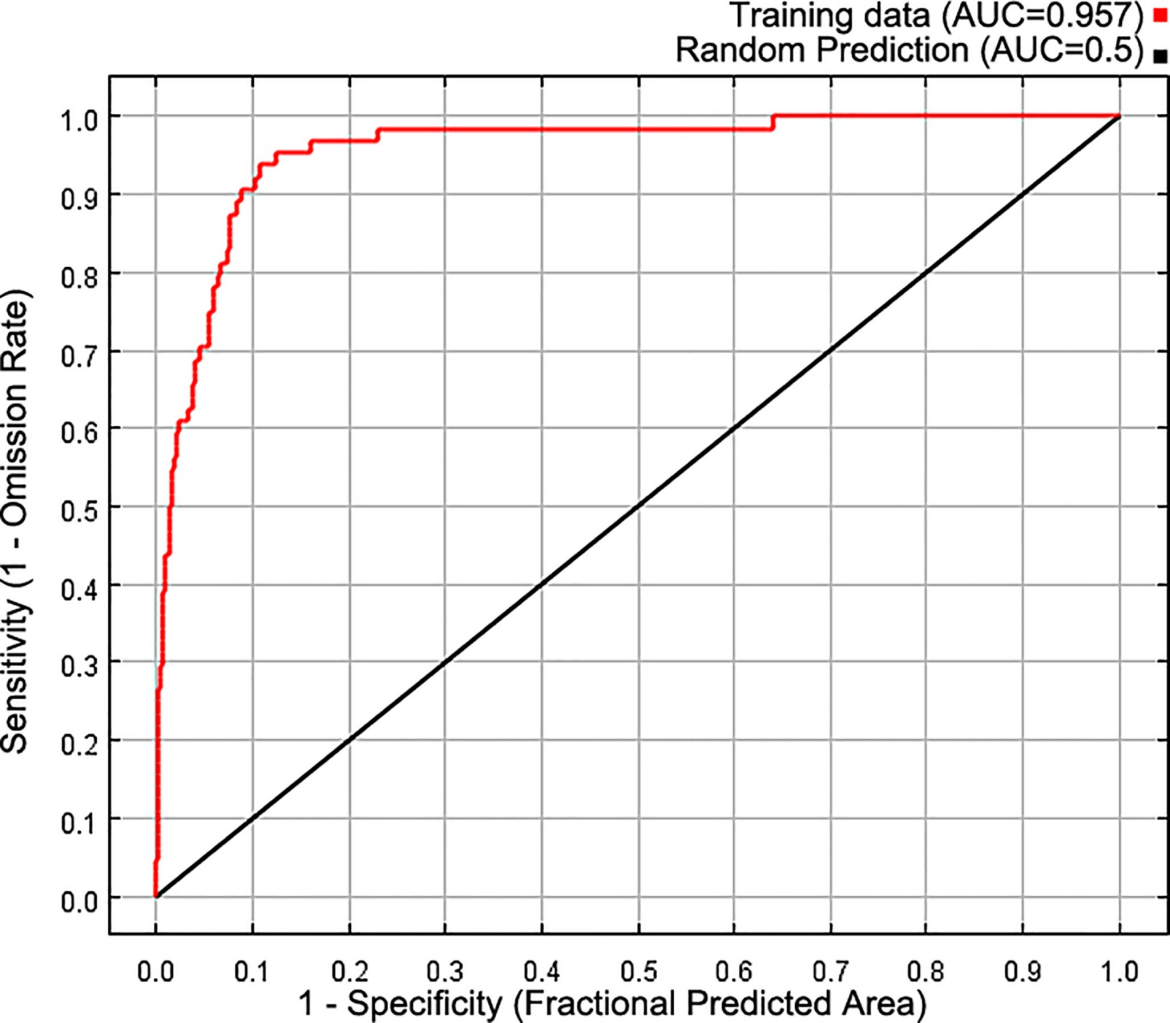
Receiver operating characteristic (ROC) curve of the MaxEnt niche model.

The niche model performed well, with an AUC value of 0.957 (Fig 1). This suggested that the method could be applied to studies on the relationship between the geographic distribution of *Sphagnum* bogs and climate change.

### Spatial-temporal shifts in the distributional pattern and areas of *Sphagnum* bogs under climate change

**Fig 2:**
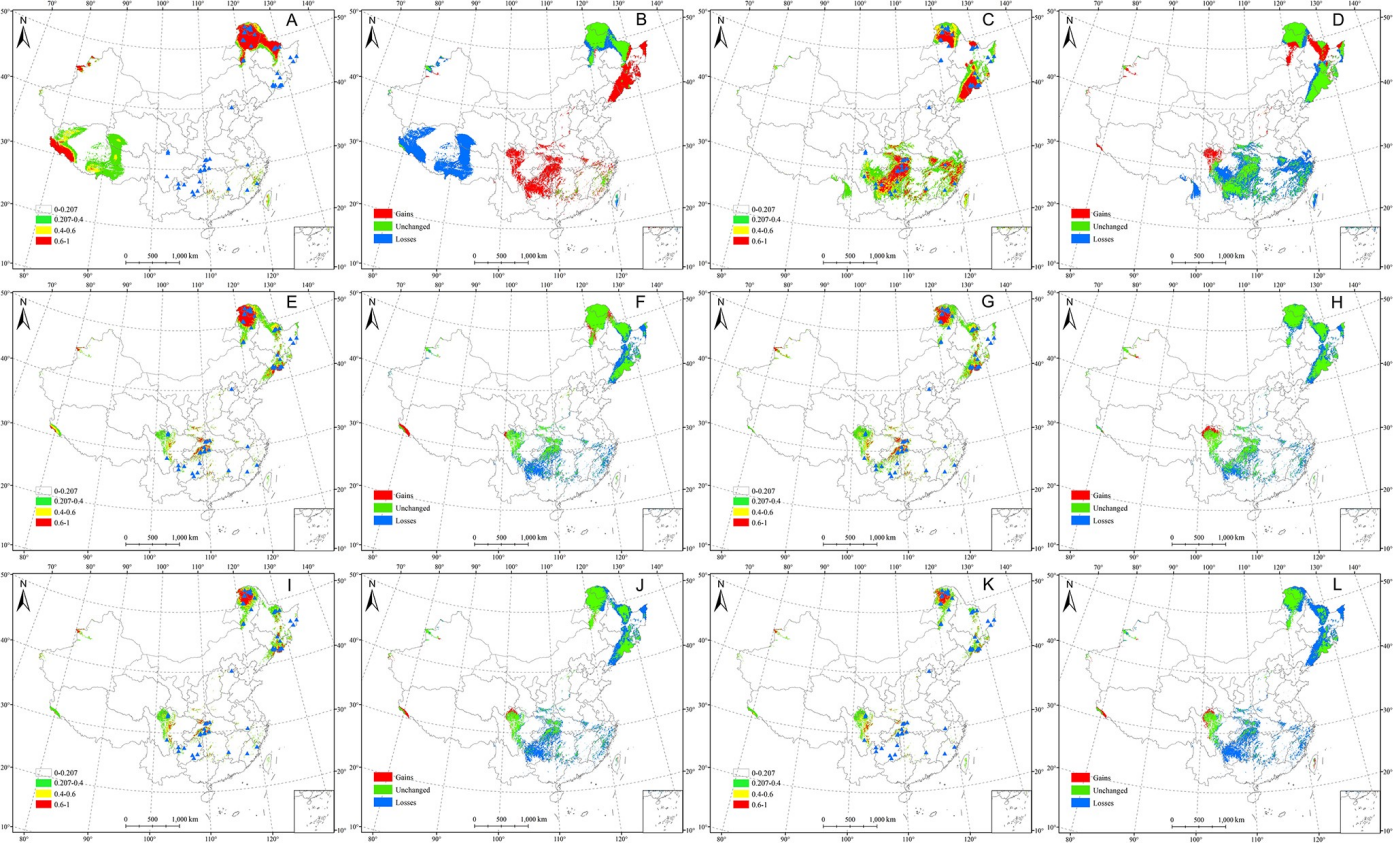
Historical and future distribution patterns of *Sphagnum* bogs in different scenarios. (A) Potential distribution pattern of *Sphagnum* bogs in the last interglacial period. (B) Comparison of the potential distribution pattern of *Sphagnum* bogs between the current distribution and the last interglacial period. (C) Potential distribution pattern of *Sphagnum* bogs in the last glacial maximum period. (D) Comparison of the potential distribution pattern of *Sphagnum* bogs between the current distribution and the last glacial maximum period. (E) Potential distribution pattern of *Sphagnum* bogs in 2050 under the RCP2.6 scenario. (F) Comparison of the potential distribution pattern of *Sphagnum* bogs between the current distribution and 2050 under the RCP2.6 scenario. (G) Potential distribution pattern of *Sphagnum* bogs in 2070 under the RCP2.6 scenario. (H) Comparison of the potential distribution pattern of *Sphagnum* bogs between the current distribution and 2070 under the RCP2.6 scenario. (I) Potential distribution pattern of *Sphagnum* bogs in 2050 under the RCP8.5 scenario. (J) Comparison of the potential distribution pattern of *Sphagnum* bogs between the current distribution and 2050 under the RCP8.5 scenario. (K) Potential distribution pattern of *Sphagnum* bogs in 2070 under the RCP8.5 scenario. (L) Comparison of the potential distribution pattern of *Sphagnum* bogs between the current distribution and 2070 under the RCP8.5 scenario.

**Fig 3:**
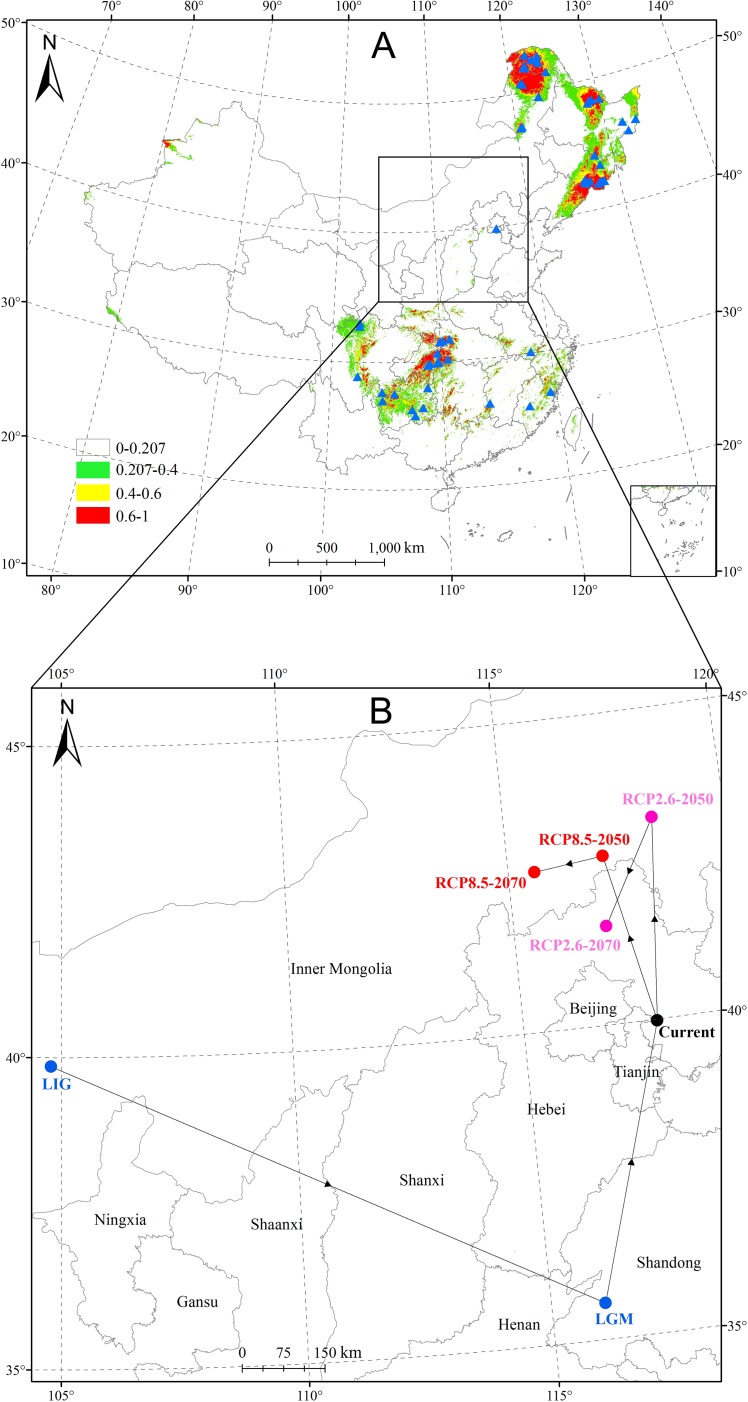
Current potential distribution pattern of *Sphagnum* bogs and migratory routes of the habitat distribution center in historical and future climate scenarios. (A) Current potential distribution patterns. (B) Migratory routes of the suitable habitat distribution center under historical and future climate scenarios. The blue dots represent the suitable habitat distribution center under the last interglacial climate scenario and the last glacial maximum climate scenario; the black dot represents the suitable habitat distribution center under the current climate scenario; the pink dots represent suitable habitat distribution centers under the RCP2.6 climate scenario in 2050 and 2070; the red dots represent the suitable habitat distribution centers under the RCP8.5 climate scenario in 2050 and 2070.

Based on current geographical distribution records and climatic data, a map predicting the potential distribution areas (suitable habitats and unsuitable habitats) for *Sphagnum* bogs was generated. A threshold of 0.207 was obtained by using the sensitivity-specificity sum maximization approach. The distribution of suitable habitat areas for *Sphagnum* bogs was divided into four categories according to suitability: (i) 0~0.207 indicated unsuitable habitats marked with no color; (ii) 0.207~0.4 indicated low-suitability areas, color-coded green; (iii) 0.4~0.6 indicated appropriate areas, color-coded yellow; and (iv) 0.6~1 indicated high-suitability areas, color-coded red (Figs 2 and 3).

The dynamic distribution included changes in scope and area. On the whole, it exhibited a discontinuous plaque pattern across China from the past to the future. The extant distribution generated by MaxEnt modeling was in accordance with actual distributional records, and the suitable habitats might occur concentrated in the following regions according to intensity: First, northeastern China including the Greater Khingan Mountains (Inner Mongolia and Heilongjiang Province), the Lesser Khingan Mountains (Heilongjiang Province) and the Changbai Mountains (Heilongjiang, Jilin, and Liaoning Province), where are mainly regions with cold, humid climates; second, southwestern China, including western Hubei Province, northern and southwestern Chongqing, most of Guizhou Province except the southern region, southern Shaanxi Province, as well as northern and southern Sichuan Province and so on; and third, eastern China, including central and northeastern Fujian Province, most of Zhejiang Province except the northern region, southwestern Anhui Province and southwestern Jiangxi Province (Fig 3A). However, the distributional pattern in LIG was different from the current, and the habitats mainly concentrated in the Greater Khingan Mountains, the Lesser Khingan Mountains, western and central Tibet, northwestern Xinjiang Province, and the sporadic distribution in east China.

In terms of changes in area compared with the current, there was a result that obtained for the loss of the original area and the gain of the new area. Currently, the total area of *Sphagnum* bogs was ca. 9.24×10^5^ km^2^. Historically, the total area in LIG was ca. 9.15×10^5^ km^2^ (1.14%), which was similar to the current. However, the total area expanded in the LGM with an area ca. 13.57×10^5^ km^2^ (-31.57%). The gain area in LIG compared to the current occurred in the northwestern Changbai Mountains and around the Sichuan Basin, with ca. 6.09×10^5^ km^2^ (66.50%)(Fig 2A and 2B, Table 2). The loss area was as high as ca. 5.98×10^5^ km^2^ (65.37%) in the LIG and ca. 6.35 ×10^5^ km^2^ (46.78%) in the LGM, respectively, which occurred around almost all the bogs (Fig 2A–2D, Table 2). Obviously, the loss area in LGM occurred in the western and central Tibet, and the northwestern Xinjiang Province. In the immediate future such as RCP2.6 scenarios, the total area reduced to ca. 5.74×10^5^ km^2^ (-61.03%) in RCP2.6–2050 (Fig 2E and 2F, Table 2) and ca. 5.87×10^5^ km^2^ (-57.57%) in RCP2.6–2070 (Fig 2G and 2H, Table 2). In the RCP8.5–2050 scenarios, the loss area increased as much as ca. 4.77×10^5^ km^2^ (-93.88%) (Fig 2I and 2J, Table 2) and reached to ca. 3.48×10^5^ km^2^ (-165.87%) in 2070 (Fig 2K and 2L, Table 2).

**Table 2:** Shifts in the potential habitat area of *Sphagnum* bogs under different climatic scenarios.

Climate scenarios	Area (×10^5^ km^2^)					Proportion of area (%)			
	Past/Future	Loss	Gain	Unchanged	Total	Loss	Gain	Unchanged	Total
**LIG**	9.15	5.98	6.09	3.18	0.10	65.37	66.50	34.78	1.14
**LGM**	13.57	6.35	2.06	7.19	-4.28	46.78	15.21	52.94	-31.57
**RCP2.6–2050**	5.74	3.90	0.39	5.38	-3.50	67.89	6.86	93.67	-61.03
**RCP2.6–2070**	5.87	3.67	0.30	5.60	-3.38	62.65	5.07	95.44	-57.57
**RCP8.5–2050**	4.77	4.71	0.24	4.56	-4.48	98.83	4.95	95.64	-93.88
**RCP8.5–2070**	3.48	6.04	0.27	3.23	-5.77	173.64	7.77	92.86	-165.87

### Migratory routes of potential distribution centers of *Sphagnum* bogs in the context of climate change

The centroid of the modern-day distribution of *Sphagnum* bogs is represented by the black dot, which is located in eastern Hebei Province (117.88E, 39.98N) (Fig 3A). The historical suitable distribution center moved from the central Inner Mongolia (104.79E, 39.86N) in LIG to the western Shandong Province (116.10E, 35.59N) in LGM, and thereafter shifted toward the current distributional centroid. The future potential centroids represented by the pink dots moved to the northwest under RCP2.6–2050 (118.35E, 43.23N) and RCP2.6–2070 (117.04E, 41.58N). Another route, that under the high-concentration greenhouse gas emission scenario RCP8.5, has the central situation represented by the red dots shifted from its current location to in 2050 (117.15E, 42.71N) and 2070 (115.59E, 42.57N) (Fig 3B). Taken together, there was a movement toward the northwest under both future emissions scenarios (RCP2.6, RCP8.5).

### Key environmental factors influencing the distribution of *Sphagnum* bogs

*Sphagnum* bog development is strongly linked to climate. The contribution rate of each environmental factor to the MaxEnt model obtained by the jackknife method is shown in Table 3. The order of the most important environmental factor influencing the distribution of *Sphagnum* bogs were Bio14 (37.3%), Bio10 (22.0%) and Bio18 (18.6%), with a total cumulative contribution rate up to 77.9%. Therefore, the abovementioned contributors were dominant factors that affected the distributional pattern of *Sphagnum* bogs.

**Table 3:** The contribution rate of each environmental factor influencing the suitable distribution of *Sphagnum* bogs.

Codes	Percent contribution (%)
**Bio14**	37.3
**Bio10**	22.0
**Bio18**	18.6
**Bio3**	8.3
**Bio6**	5.3
**Bio8**	3.5
**Bio7**	1.9
**Bio2**	1.2
**Bio11**	0.7
**Bio4**	0.4
**Bio17**	0.2
**Bio15**	0.2
**Bio19**	0.1
**Bio12**	0.1
**Bio13**	0.1
**Bio5**	0.1
**Bio9**	0.0
**Bio1**	0.0
**Bio16**	0.0

**Fig 4:**
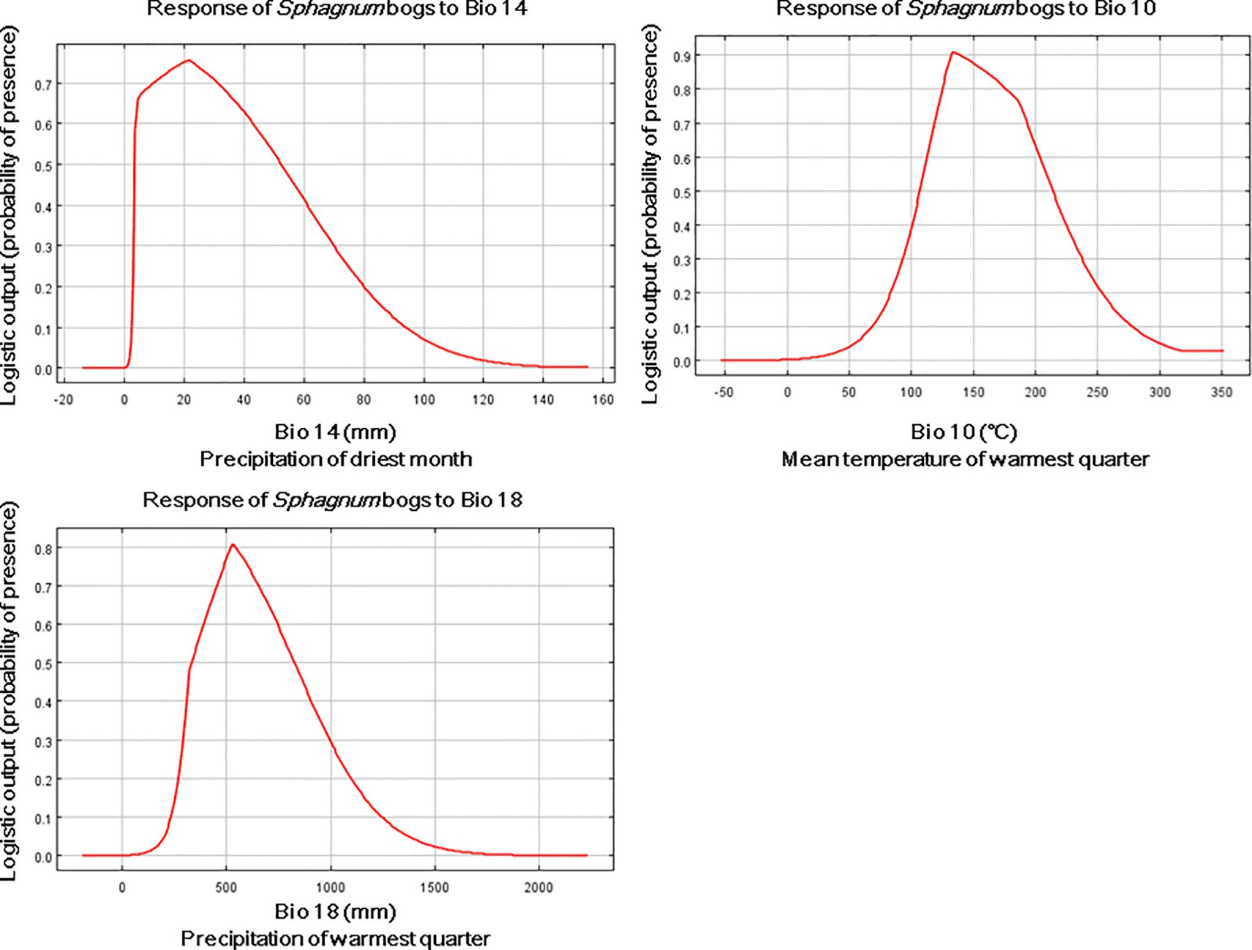
Response curves for key environmental predictors in the species distribution model for *Sphagnum* bogs. Bio14 is the precipitation of driest month; Bio10 is the mean temperature of warmest quarter; Bio18 is the precipitation of warmest quarter.

The responses of the three key environmental factors and their respective thresholds are shown in Fig 4. Response curves pointed out the threshold ranges of the environmental parameters: precipitation of driest month (Bio14) ranged from 10 to 75 mm, mean temperature of warmest quarter (Bio10) ranged from 6 to 22.5 °C, and precipitation of warmest quarter (Bio18) ranged from 300~1050 mm.

## Discussion

The evaluation index of the area under the curve of the receiver model was used, and the predictive performance AUC value was 0.957. The results revealed high precision, which indicated that the results were reliable and accurate for the prediction of *Sphagnum* bog distributional patterns. Our study verified the hypothesis, namely, that changes in distribution patterns under ancient climate conditions could provide an effective way to predict distribution areas under future climate change [37].

The prediction maps objectively reflected that the spatial distribution pattern of *Sphagnum* bogs had formed in a fragmented and sparse way since LIG. Then, the distribution pattern was similar to the current since the LGM, accompanied by the disappearance of suitable habitats areas in western and central Tibet, and the northwestern Xinjiang Province. Our findings revealed that the suitable habitats were mainly northeastern, southwestern and eastern China in turn. Simulated research nationally notes that *Sphagnum* bogs usually settle in the northern hemisphere and range from temperate zones to northern polar regions, such as Alaska, Canada, and Siberia [38], which confirms that wet and cold climate benefits the bogs. It is noticeable that the northeastern region is located in the most northern part of China, where plants distribute discretely are more susceptible to the environment. Gunnarsson reports that 45°N is one of the two productivity peaks of the net primary production of peatlands, where the distribution areas of the *Sphagnum* bogs in the northeast China of this study were located [39]. Hence, it is necessary to monitor and implement protection for *Sphagnum* bogs, particularly in northeastern China, because this area is the largest suitable area in China for *Sphagnum* bogs [40].

Historically, the total area of *Sphagnum* bogs in LIG was almost the same as the current but got an increase in LGM. It is presumably due to the similar climate conditions to the current in LIG. While the substantial drop in temperature during the ice age in LGM built a cold and wet environmental atmosphere. During this time, it was cold and wet to support extensive paludification with more local *Sphagnum* bogs that have been recorded and formed the distribution pattern similar to the current. Unfortunately, the geographical distribution of *Sphagnum* bogs was limited, and the reserves in existence today were still rare then overall. As expected, the total suitable area reduced in the future climate scenarios, especially in RCP8.5, which suggested that global warming would hinder the development of *Sphagnum* bogs seriously. Conversely, the different conclusion was drawn in North America [41]. The reason, as described by Gunnarsson, might be that climatic parameters together with geographical position are important for *Sphagnum* bogs. The precipitation in much of North America will increase in the immediate future, which is different in China [39]. Another researcher Jun Cheng comes to the conclusion that climate warming will widen the distribution of the *Calymperes* [42]. It might be because the *Calymperes* originated in the tropics, while the *Sphagnum* plants were colder origins and preferred cool and wet climatic conditions. Therefore, under the background of future climate warming, a warm and dry climate is unsuitable for the development of the bogs. Additionally, our study also found that the edges of the bogs were sensitive to ongoing climate change, with the gain and loss areas all occurring on the margins of bogs, especially in RCP8.5. After deglaciation, the margins were identified as being at a high risk of disappearing under future climate scenarios and should be protected in advance. The models showed that, in the immediate future, the total suitable area for *Sphagnum* bogs could get a large-scale reduction. Collectively, these results implied that climate change might pose a great risk to the bogs and that there might be a stronger climatic in the future.

The expansion-contraction model describes vegetation dynamics in the Late Quaternary with the core argument that the population migrates from south to north in LIG but shows the opposite trend in LGM [43]. It is thus clear that our findings were consistent with the hypothesis of the expansion-contraction model. Generally, climate warming causes the distribution area of many plant species to shift northward in the Northern Hemisphere [44] and the center of distribution shifts along with climate change. Jun Cheng reports that the *Calymperes* migrates to the north accompanying climate warming [42]. Gignac simulates the distribution of mosses in a marsh in Mackenzie Valley in Canada and concludes that mosses move northward as a result of elevated temperatures and drought [45]. Similar to the studies above, bogs migrated to the northwest in future warming scenarios. We further discovered that the ability of bryophytes to migrate was weaker than that of other higher plants, which might due to their spores only spread by water over short distances.

Climatic factors are driving forces for bog development [46] because climate change triggers the reallocation of precipitation and heat over large spatial-temporal scales [47,48]. Only at a certain humidity and temperature conditions can *Sphagnum* bogs develop well. Our results revealed that the determinant factors affecting distribution were, in order, Bio14, Bio10 and Bio18. In detail, Bio14, and Bio18 are related to moisture. As an extreme climate factor, Bio14, precipitation of driest month, increased the distributional variation. The water supply of *Sphagnum* plants is derived from atmospheric precipitation, and it is likely to be difficult for *Sphagnum* plants to survive due to desiccation during the dry summers in places such as Tasmania and Victoria [48]. Bio18, precipitation of warmest quarter, was also a major environmental factor for potential habitats in most similar studies [19]. In other words, therefore, aridity might prevent the development and spread of the bogs. Distribution pattern maps of bogs likewise showed that there was no occurrence of bogs in arid regions but that they settled in cold-temperate areas with high-humidity zones where the water accumulation exceeded the evaporation. Hence, water was considered a dominant restriction factor for the distribution of *Sphagnum* bogs.

The factors currently influencing the survival of *Sphagnum* bogs, similar to those affecting Australasia, are primarily global warming and human activities [49]. Sure enough climate change is expected to have strong negative effects on *Sphagnum* bogs according to our findings. Of particular concern is that non-climatic factors, i.e., human activities contributed to a severe loss of suitable area. There is growing evidence that human activities and populations are the most direct and strongest destructive driving factor leading to the bogs suffering a collapse and have substantially altered the original ecological balance [50,51]. For example, in northeastern China, a transformation from *Sphagnum* bogs to forestry production has changed the area from a net carbon sink to a significant carbon source [52]. Thus, it follows that human activities should be listed as the first factor threatening the survival of plants.

*Sphagnum* bogs play an extremely important role in slowing down the greenhouse effect. Once they were ruined, it is hard to restore them to the original state. Consequently, it is improvident to sacrifice ecological resources for economic value. Given this, Indonesia, Peru, and the Republic of Congo have joined the Global Peatlands Initiative to encourage international organizations and academic institutions to protect *Sphagnum* bogs. The European Union, Ireland, and Scotland have enacted laws to restrict the use of *Sphagnum* bogs. Currently, China must pay attention to protecting the precious national natural resources. For this purpose, we propose three management strategies in response to future climate change. First, many *Sphagnum* bogs are generally neglected in field investigations because they are indistinguishable from grassy slopes. Researchers should focus on identifying and labeling *Sphagnum* bogs in field surveys and establishing new nature reserves for *in situ* conservation. Second, managers should pay attention to the borders of the bogs and take protective measures to enlarge the boundaries of nature reserves. Third, human activities that destroy *Sphagnum* bogs should be prevented.

## Conclusions

As described above, our study used large-scale bioclimatic environmental datasets to carry out quantitative simulation analyses on habitat changes in *Sphagnum* bogs across China and framed the results in a larger spatial and temporal context. This method was feasible and practical for evaluating other types of wetlands. Maps showed that *Sphagnum* bogs were distributed sparsely with few reserves and were mainly found in northeastern, southwestern and eastern China. The contour of the present distributional pattern had formed since the LGM, and it was consistent with the current distribution. In historical climate scenarios, the total area was almost the same with the current area because of the similar climate conditions in LIG, but got an increase in LGM because of the suitable cold and wet environmental atmosphere; what’s worse, the total area of *Sphagnum* bogs reduced dramatically in the future scenarios, especially in RCP8.5 scenarios. Furthermore, the boundaries of the bogs were sensitive to global warming. The migratory routes demonstrated that the centers of bog ecosystems might migrate to the northwest in the future. Environmental factors affected suitability, and water was the most determinant factor. Although future climate warming will cut suitable habitats, human activities maybe also the most influential destructive factor to accelerate the degradation of the bogs. The destruction of *Sphagnum* bogs by human activities should be stopped immediately. Through forecasting, our work provides a scientific basis for the management and protection of *Sphagnum* bogs and a warning to protect these fragile bryophytes.

## Supporting information

S1 Dataset Records of *Sphagnum* bog central points used for ecological modeling. (CSV)
